# Cancer Burden and Inflammaging Among People Living with HIV: An Inflammatory Biomarker Analysis from an Italian Cohort

**DOI:** 10.3390/cancers18182929

**Published:** 2026-09-10

**Authors:** Daniela Segala, Bianca Maria Interlenghi, Rosario Cultrera

**Affiliations:** 1 Department of Translational Medicine and for Romagna, University of Ferrara, Azienda Unità Sanitaria Locale di Ferrara, 44121 Ferrara, Italy; rosario.cultrera@unife.it; 2 Faculty of Medicine, Pharmacy and Prevention, University of Ferrara, 44121 Ferrara, Italy; biancama.interlenghi@edu.unife.it

**Keywords:** HIV, cancer, chronic inflammation, inflammaging, inflammatory biomarkers, non-AIDS-defining cancers

## Abstract

**Background/Objectives:** Despite effective antiretroviral therapy, cancer remains a relevant comorbidity in people living with HIV (PLWH), with an increasing burden of non-AIDS-defining cancers (NADCs). We assessed cancer prevalence and systemic inflammation using a composite score based on routine inflammatory biomarkers. **Methods:** We conducted a retrospective cross-sectional study of PLWH in Ferrara, Italy. Cancer history, immunovirological parameters, IL-6, CRP, D-dimer, and monocyte counts were collected. A composite inflammatory score (0–4) was derived from biomarker abnormalities and compared between AIDS-defining cancers (ADCs) and NADCs. **Results:** Among 566 participants, 64 had a history of cancer (11.3%; 95% CI, 8.7–13.9%), accounting for 79 diagnoses: 23 (29.1%) ADCs and 56 (70.9%) NADCs. Most diagnoses (82.3%) were associated with inflammatory scores of 0–1, whereas 17.7% had scores of 2–4, with similar distributions between ADCs and NADCs (17.4% vs. 17.9%; *p* = 0.962). D-dimer elevation was more frequent among NADCs than ADCs (42.9% vs. 21.7%). Higher inflammatory scores were significantly associated with lower current CD4+ T-cell counts (*p* = 0.043). **Conclusions:** Cancer was a substantial comorbidity among PLWH, with NADCs predominating despite favorable immunovirological status. The composite score integrated routinely available inflammatory, coagulation, and immune parameters, with higher scores associated with lower CD4+ counts. Although inflammatory profiles did not differ significantly between ADCs and NADCs, this finding highlights the complexity of immune dysregulation in PLWH and the limitations of individual circulating biomarkers. Further validation of composite inflammatory scores may provide a clinically accessible framework for characterizing residual immune dysfunction and its relationship with cancer development and outcomes.

## Simple Summary

People living with HIV are living longer because of effective antiretroviral therapy, but cancer remains an important health concern. We investigated cancer prevalence and systemic inflammation in an Italian cohort of people living with HIV. In this Italian cohort, 11.3% of patients had a history of cancer, and most recorded diagnoses were non-AIDS-defining cancers. Cancer occurred despite generally effective virological control and satisfactory current CD4+ T-cell counts. Most cancer diagnoses were associated with absent or mild systemic inflammatory abnormalities based on the available biomarker measurements, and inflammatory profiles did not differ significantly between AIDS-defining and non-AIDS-defining cancers, although greater inflammation was associated with lower CD4+ T-cell counts. These findings suggest that routine inflammatory biomarkers alone may not fully capture cancer susceptibility in people living with HIV and reinforce the importance of cancer prevention and surveillance as part of long-term HIV care.

## 1. Introduction

Human immunodeficiency virus (HIV) infection remains a major global public health challenge, with approximately 41.0 million people living with HIV worldwide in 2025 [1]. The introduction of combination antiretroviral therapy (cART) substantially reduced HIV-related morbidity and mortality, transforming HIV infection into a manageable chronic condition [2]. Improved virological suppression, immune recovery, and survival have led to progressive aging of people living with HIV (PLWH) and a shift in disease burden toward chronic noncommunicable comorbidities, including cancer. Although AIDS-defining cancers (ADCs) have declined markedly in the cART era, non-AIDS-defining cancers (NADCs) account for an increasing proportion of malignancies, and overall cancer incidence among PLWH remains approximately 1.6–1.7 times higher than in the general population [3].

Cancer development in PLWH likely reflects the interaction of aging, persistent immune dysregulation, oncogenic viral coinfections, behavioral and environmental factors, and prolonged HIV infection. Despite sustained virological suppression and CD4+ T-cell recovery, persistent immune activation and chronic low-grade inflammation may continue. Proposed mechanisms include microbial translocation, persistence of the viral reservoir, low-level viral protein expression, monocyte and endothelial activation, and premature immune senescence [4,5,6]. These processes contribute to inflammaging, which may accelerate biological aging, impair antitumor immune surveillance, and promote oxidative stress, genomic instability, and malignant transformation.

Circulating inflammatory and coagulation biomarkers may provide clinically relevant information on these persistent alterations. Interleukin-6 (IL-6) reflects cytokine-mediated immune activation, C-reactive protein (CRP) systemic inflammation, and D-dimer coagulation and endothelial activation [7]. However, whether routinely available biomarkers can capture the complex immune–inflammatory alterations associated with cancer in treated PLWH remains unclear, particularly across different cancer categories. ADCs remain closely associated with severe or historical immunodeficiency and oncogenic viral infections, whereas NADCs comprise a heterogeneous group associated with aging, long-term immune dysregulation, chronic inflammation, viral coinfections, and conventional cancer risk factors [8]. Integrating markers reflecting complementary inflammatory, coagulation, and immune pathways into a composite score may therefore provide a more comprehensive and clinically accessible measure of systemic inflammatory burden. However, evidence remains limited on whether these routinely available biomarkers can be combined into a simple composite measure to characterize residual systemic inflammation among PLWH with cancer and to compare inflammatory patterns across cancer categories.

This study represents the first investigation within the Ferrara HIV cohort to integrate cancer burden with residual systemic inflammation. Building on previous characterization of cancer prevalence in this population [9], we applied an exploratory composite inflammatory score to explore whether systemic inflammatory patterns differ between ADCs and NADCs and how they relate to immunovirological status. This exploratory approach was designed to provide a pragmatic framework for characterizing current residual systemic inflammation among PLWH with a history of cancer, and to generate hypotheses regarding the potential contribution of composite inflammatory profiles to cancer susceptibility. However, because the study did not include a cancer-free comparison group in the cancer-related analyses and biomarkers were measured cross-sectionally, it was not designed to establish a predictive association with cancer risk.

## 2. Materials and Methods

A retrospective cross-sectional study was conducted among PLWH receiving care at the HIV/AIDS Unit of Ferrara University Hospital from January to December 2024. Adults aged ≥18 years with documented HIV-1 or HIV-2 infection, receiving antiretroviral therapy, and with at least one routine laboratory assessment during the study period were eligible. Data for at least two of the three inflammatory biomarkers (IL-6, CRP, and D-dimer) were required. Patients with insufficient clinical or laboratory data were excluded. For cancer-related analyses, only malignancies diagnosed after HIV diagnosis were considered.

Demographic, clinical, immunovirological, and cancer data were retrieved from medical records and included age, sex, nationality, CDC clinical classification, duration of HIV infection, cancer history, plasma HIV RNA, CD4+ T-cell and monocyte counts, IL-6, CRP, and D-dimer. Malignancies were classified as ADCs or NADCs according to standard criteria.

Laboratory parameters were obtained from the most recent routine assessment performed during the 2024 study period. These measurements were collected cross-sectionally and did not necessarily coincide with, or precede, the diagnosis of cancer. Accordingly, the inflammatory biomarkers and the derived composite score were intended to characterize current systemic inflammatory status rather than inflammation at the time of cancer development. Plasma HIV RNA was quantified by real-time polymerase chain reaction (lower limit of detection, 20 copies/mL) and categorized as target not detected, <20, 20–49, or ≥50 copies/mL. CD4+ T-cell and monocyte counts were determined by multiparameter flow cytometry. Elevated inflammatory biomarkers were defined according to laboratory thresholds: IL-6 ≥ 6.40 pg/mL, CRP ≥ 0.50 mg/dL, and D-dimer ≥ 0.50 µg/mL fibrinogen-equivalent units; the reference range for monocytes was 200–1000 cells/µL.

A composite inflammatory score was developed to characterize systemic inflammatory burden. One point was assigned for each abnormal parameter: IL-6 ≥ 6.40 pg/mL, CRP ≥ 0.50 mg/dL, D-dimer ≥ 0.50 µg/mL, and monocyte count <200 or >1000 cells/µL, yielding a score from 0 to 4. The cutoffs corresponded to the reference ranges used by the local clinical laboratory. Each parameter was given equal weight to preserve a simple additive structure, as no validated weighting scheme was available for this exploratory score. The score was analyzed both ordinally and dichotomized as 0–1 (absent/mild inflammatory abnormalities) or 2–4 (significant inflammatory abnormalities). Cancer diagnoses with any missing value among the parameters included in the composite inflammatory score were excluded from the score calculation.

Cancer prevalence was calculated at the patient level as the proportion of participants with at least one eligible malignancy, with 95% confidence intervals. Because some participants had multiple malignancies, cancer-type analyses were performed at the diagnosis level. Demographic, clinical, immunovirological, and inflammatory characteristics were therefore repeated for each eligible cancer diagnosis, with patient identification code used as the clustering variable to account for within-patient correlation.

Categorical variables were summarized as frequencies and percentages. Associations between dichotomized inflammatory status and demographic, clinical, immunovirological, and cancer-related characteristics were assessed using the Rao–Scott-adjusted chi-square test. Statistical analyses were performed using R, version 4.3.1 (R Foundation for Statistical Computing, Vienna, Austria). Statistical significance was defined as a two-sided *p* < 0.05. Data were anonymized before analysis.

## 3. Results

A total of 684 patients were assessed for eligibility; after excluding 118 with incomplete data, 566 were included in the analysis. The cohort comprised 393 males (69.4%) and 173 females (30.6%), with 470 participants (83.0%) of Italian nationality. Most participants were aged 46–65 years (n = 356; 62.8%), 96 (17.0%) were >65 years, and 428 (75.6%) had been living with HIV for >10 years.

Most participants had effective virological control: 288 (50.9%) had undetectable HIV RNA, 175 (30.9%) had detectable HIV RNA < 20 copies/mL, 32 (5.7%) had 20–49 copies/mL, and 41 (7.2%) had ≥50 copies/mL; viral-load data were unavailable for 30 (5.3%). CD4+ T-cell counts were >500 cells/µL in 448 participants (79.1%), 200–500 cells/µL in 91 (16.1%), and <200 cells/µL in 27 (4.8%). Elevated IL-6, CRP, and D-dimer levels were observed in 89 (15.7%), 126 (22.2%), and 107 participants (18.9%), respectively, while monocyte counts were within the reference range in 549 (97.0%) (Table 1).

Among the 566 participants, 64 had a history of cancer, corresponding to a prevalence of 11.3% (95% CI, 8.7–13.9%). Of these, 47 (73.4%) were male, 56 (87.5%) were Italian, 44 (68.8%) were aged 46–65 years, and 16 (25.0%) were >65 years; 51 (79.7%) had been living with HIV for >10 years. Among participants with a history of cancer, 31 (48.4%) had undetectable HIV RNA, 20 (31.3%) had <20 copies/mL, 3 (4.7%) had 20–49 copies/mL, and 5 (7.8%) had ≥50 copies/mL; data were unavailable for 5 (7.8%). CD4+ T-cell counts were >500 cells/µL in 43 participants (67.2%), 200–500 cells/µL in 15 (23.4%), and <200 cells/µL in 6 (9.4%). Elevated IL-6, CRP, and D-dimer levels were observed in 15 (23.4%), 16 (25.0%), and 17 participants (26.6%), respectively.

Overall, 79 cancer diagnoses were recorded among the 64 participants with a history of cancer: 23 (29.1%) were ADCs and 56 (70.9%) NADCs. Kaposi sarcoma was the most frequent ADC (n = 18; 22.8% of all cancer diagnoses), followed by Burkitt lymphoma (n = 3; 3.8%) and diffuse large B-cell lymphoma (n = 2; 2.5%). Among NADCs, skin cancers were the most common (n = 25; 31.6%), followed by head and neck (n = 9; 11.4%), thoracic (n = 8; 10.1%), genitourinary (n = 5; 6.3%), hematopoietic and lymphatic (n = 4; 5.1%), and gastrointestinal malignancies (n = 3; 3.8%); neuroendocrine and central nervous system malignancies accounted for one diagnosis each (1.3%) (Table 2).

Among ADC diagnoses, 20 (87.0%) occurred in males and 21 (91.3%) in Italian participants. Most occurred at age 46–65 years (n = 13; 56.5%) or >65 years (n = 7; 30.4%), and 15 (65.2%) in participants living with HIV for >10 years. CDC stages C3 (n = 13; 56.5%) and C2 (n = 7; 30.4%) predominated. Current CD4+ T-cell counts were >500 cells/µL for 17 diagnoses (73.9%), 200–500 cells/µL for three (13.0%), and <200 cells/µL for three (13.0%).

Among NADC diagnoses, 40 (71.4%) occurred in males and 50 (89.3%) in Italian participants. Most occurred at age 46–65 years (n = 33; 58.9%) or >65 years (n = 21; 37.5%), and 50 (89.3%) in participants living with HIV for >10 years. CDC classification was more heterogeneous, with C2 (28.6%), C3 (19.6%), and A2 (16.1%) as the most frequent categories. Current CD4+ T-cell counts were >500 cells/µL for 39 diagnoses (69.6%), 200–500 cells/µL for 14 (25.0%), and <200 cells/µL for 3 (5.4%). D-dimer elevation was more frequent among NADCs than ADCs (42.9% vs. 21.7%), whereas elevated IL-6 was observed in 19.6% versus 26.1% and elevated CRP in 17.9% versus 26.1%, respectively.

Among the 79 cancer diagnoses, 36 (45.6%) had an inflammatory score of 0 and 29 (36.7%) a score of 1; scores of 2, 3, and 4 occurred in seven (8.9%), five (6.3%), and two diagnoses (2.5%), respectively. Overall, 65 diagnoses (82.3%) had absent or mild inflammatory abnormalities (scores 0–1), whereas 14 (17.7%) had significant abnormalities (scores 2–4). Scores of 2–4 occurred with similar frequency among ADCs and NADCs (17.4% vs. 17.9%), while scores of 0–1 occurred in 82.6% and 82.1%, respectively.

Across individual score categories, score 0 predominated among ADCs (n = 13; 56.5%), followed by score 1 (n = 6; 26.1%), score 3 (n = 2; 8.7%), and scores 2 and 4 (n = 1 each; 4.3%). Among NADCs, scores 0 and 1 were equally frequent (n = 23 each; 41.1%), followed by score 2 (n = 6; 10.7%), score 3 (n = 3; 5.4%), and score 4 (n = 1; 1.8%). Scores of 0–1 occurred in 16 of 18 Kaposi sarcoma diagnoses (88.9%) and 22 of 25 non-AIDS-defining skin cancers (88.0%). Among eight lymphoma diagnoses, five (62.5%) had scores of 0–1 and three (37.5%) had scores of 2–4; among the remaining 28 NADCs, 22 (78.6%) had scores of 0–1 and 6 (21.4%) had scores of 2–4.

Inflammatory status was significantly associated with current CD4+ T-cell count (*p* = 0.043). Scores of 2–4 occurred in three of six diagnoses with CD4+ T-cell counts <200 cells/µL, five of seventeen with counts of 200–500 cells/µL, and six of fifty-six with counts >500 cells/µL. No significant associations were observed with sex (*p* = 0.123), geographical origin (*p* = 0.693), age group (*p* = 0.775), or duration of HIV infection (*p* = 0.103) (Table 3). Inflammatory status was also not associated with cancer category (ADC vs. NADC; *p* = 0.962) (Table 4) or pathological group (lymphomas, Kaposi sarcoma, non-AIDS-defining skin cancers, and other NADCs; *p* = 0.357) (Table 5). All diagnosis-level comparisons accounted for within-patient correlation using the patient identification code as the clustering variable.

A comparative analysis was conducted to investigate the potential association between the inflammatory score (divided into two groups: absent/mild and significant inflammation) and the specific neoplastic categories ADC and NADC. The results indicate no statistically significant relationship between the magnitude of the inflammatory score and the type of neoplasm. The assessment was performed using the Rao–Scott-adjusted chi-square test (*p* = 0.962).

To assess the possible correlation between the different pathological groups and the degree of inflammation, divided into two categories (absent/mild and significant), the association between the assigned score category and the corresponding pathological group was analyzed. Although statistical significance was not reached, the analysis highlighted distributional trends of interest among the different groups. This result was confirmed using the Rao–Scott-adjusted chi-square test (*p* = 0.357).

## 4. Discussion

The advent of combination antiretroviral therapy has transformed HIV infection into a manageable chronic condition, substantially increasing life expectancy. However, persistent immune activation and low-grade inflammation, commonly described as inflammaging, remain relevant because of their potential contribution to noncommunicable comorbidities, including cancer. The decline in ADCs has been accompanied by an increasing burden of NADCs, while overall cancer incidence remains higher among PLWH than in the general population [3]. This residual risk likely reflects the combined effects of historical immunosuppression, oncogenic viral coinfections, behavioral factors, aging, and impaired immune surveillance [4]. These findings are consistent with our previous characterization of cancer burden in the Ferrara HIV cohort [9] and reinforce the growing importance of cancer prevention and surveillance in long-term HIV care.

In the present study, 64 of 566 participants had a history of cancer, corresponding to a prevalence of 11.3%. This estimate cannot be directly compared with incidence rates from population-based studies because our analysis included any eligible malignancy diagnosed after HIV diagnosis. Nevertheless, it confirms a substantial oncological burden among treated PLWH. Most participants with a history of cancer were male, largely reflecting the sex distribution of the underlying cohort. Biological sex may also influence chronic immune activation, as women generally mount stronger innate and adaptive antiviral responses through hormonal and X-linked mechanisms [10,11]; however, the limited number of women precludes conclusions regarding sex-specific cancer or inflammatory profiles.

Among 79 cancer diagnoses, 23 (29.1%) were ADCs and 56 (70.9%) were NADCs. This predominance of NADCs is consistent with contemporary epidemiological trends associated with improved survival and progressive aging in the cART era. Park et al. reported an approximately 50% higher overall cancer risk among PLWH than in the general Korean population [12], while the Italian ICONA cohort showed persistently elevated virus-associated malignancies and increasing age at cancer diagnosis over time [3]. In our cohort, skin cancers were the most frequent NADCs, followed by head and neck, thoracic, genitourinary, and hematopoietic and lymphatic malignancies. Moreover, 89.3% of NADC diagnoses occurred in individuals living with HIV for >10 years and most after age 45, supporting the potential cumulative contribution of aging, prolonged HIV infection, conventional cancer risk factors, and persistent immune dysregulation. Long-term exposure to older antiretroviral regimens may additionally contribute through oxidative stress, mitochondrial dysfunction, and accelerated cellular senescence [13], although these mechanisms were not directly assessed.

ADCs were predominantly represented by Kaposi sarcoma and aggressive B-cell lymphomas. Persistent oncogenic viral infections, including human herpesvirus 8 and Epstein–Barr virus, together with severe or historical immunodeficiency, remain major drivers of these malignancies [8]. The predominance of CDC stages C2 and C3 among ADCs, compared with a more heterogeneous distribution among NADCs, supports the relevance of patients’ immunological history even after virological suppression and quantitative immune recovery. Because CDC classification incorporates both historical CD4+ T-cell strata and prior AIDS-defining conditions, it was used as a categorical indicator of previous immunological damage; the exact CD4+ T-cell nadir was therefore not analyzed separately to avoid introducing a substantially overlapping variable. Previous uncontrolled viremia and low CD4+ T-cell nadir may leave persistent immunological or epigenetic effects influencing subsequent cancer susceptibility [14].

Notably, most participants showed satisfactory current immunovirological control. CD4+ T-cell counts were >500 cells/µL in 79.1% of the overall cohort and 67.2% of participants with a history of cancer and were similarly preserved in most ADC and NADC diagnoses. Conversely, CD4+ T-cell counts <200 cells/µL were more frequent among ADCs. These findings are consistent with a stronger relationship between ADCs and severe or historical immune impairment, while NADCs frequently occur despite preserved current immune status. Because CD4+ T-cell counts were measured in 2024 rather than at cancer diagnosis, they cannot reflect immune status at tumor development; nevertheless, they illustrate the limitations of cross-sectional immunovirological parameters in representing cumulative oncological vulnerability.

The analysis of IL-6, CRP, D-dimer, and monocyte counts provided complementary information on systemic inflammatory status at the time of the 2024 assessment. However, because these biomarkers were measured cross-sectionally and generally after cancer diagnosis, they cannot indicate whether systemic inflammation preceded cancer development or was present at the time of tumor diagnosis. The observed profiles may instead reflect current residual immune dysfunction and may also be influenced by cancer-related factors, treatment, comorbidities, or other intercurrent conditions. Therefore, the findings should not be interpreted as evidence of a temporal relationship between inflammation and cancer development. Elevated IL-6, CRP, and D-dimer levels were observed in approximately one-fifth to one-quarter of participants with a history of cancer, whereas monocyte counts were almost universally within the reference range. This may indicate that persistent monocyte activation is predominantly functional rather than quantitative; soluble markers such as sCD14 and sCD163 may better capture monocyte/macrophage activation [15].

D-dimer elevation was descriptively more frequent among NADCs than ADCs (42.9% vs. 21.7%). D-dimer reflects coagulation and endothelial activation and has been associated with serious non-AIDS morbidity and mortality [7]. More recent studies have also reported associations between inflammatory/coagulation biomarkers and NADCs despite sustained virological suppression [16,17]. Our findings are therefore compatible with a contribution of low-grade coagulation and endothelial activation to the biological environment associated with NADCs. However, this difference was not independently tested and should remain descriptive rather than being interpreted as evidence of a specific association.

A central aspect of this study was the application of a composite inflammatory score integrating routinely available inflammatory, coagulation, and immune parameters. Most cancer diagnoses showed minimal systemic abnormalities. Higher scores occurred with almost identical frequency among ADCs and NADCs (17.4% vs. 17.9%; *p* = 0.962). Thus, the score did not distinguish the two cancer categories, suggesting that conventional circulating biomarkers may capture shared systemic inflammatory processes rather than cancer-specific inflammatory phenotypes. However, given the limited subgroup sizes, this interpretation remains exploratory and does not establish biological equivalence between ADCs and NADCs.

The distribution of individual score categories nevertheless provides exploratory biological insights. ADCs were most frequently associated with score 0, whereas NADCs were equally distributed between scores 0 and 1. This pattern may be compatible with a stronger contribution of historical immune impairment and oncogenic viral persistence to ADCs, while low-grade immune, endothelial, or coagulation activation may contribute to NADCs. These observations should not be interpreted as evidence of distinct pathogenetic mechanisms because the overall comparison was not significant. More broadly, the predominance of scores 0–1 suggests that routinely measured circulating biomarkers may not fully capture inflammatory pathways relevant to carcinogenesis. Microbial translocation, low-level viral protein expression, oxidative stress, and tissue-specific inflammation may persist despite virological suppression and normal circulating biomarkers [5,18,19].

Inflammatory patterns also varied descriptively across pathological groups. Lymphomas showed the broadest score distribution, with scores of 2–4 in three of eight diagnoses, compared with two of eighteen Kaposi sarcomas and three of twenty-five non-AIDS-defining skin cancers. Systemic cytokine activation, tumor-related inflammation, or disease burden may contribute to higher scores in lymphomas, although the small number of cases precludes diagnostic or prognostic interpretation. Kaposi sarcoma and non-AIDS-defining skin cancers were predominantly associated with scores of 0–1, potentially reflecting localized or compartmentalized mechanisms not captured by circulating biomarkers. Thoracic malignancies were also confined to scores of 0–1, whereas head and neck cancers showed a broader distribution, consistent with their multifactorial etiology involving smoking, alcohol, oncogenic viruses, and chronic inflammatory injury [20,21,22,23]. These subgroup observations remain exploratory. This analysis partially addressed the heterogeneity of the NADC category, while further stratification according to viral association would require larger subgroup sizes.

Current CD4+ T-cell count was the only variable significantly associated with inflammatory status (*p* = 0.043), with higher inflammatory scores occurring more frequently at lower CD4+ T-cell counts. This relationship is biologically plausible, as advanced immunodeficiency may coexist with microbial translocation and greater immune activation. However, the small number of observations in the lowest CD4+ category and absence of multivariable adjustment require caution. No significant associations were identified with sex, geographical origin, age, duration of HIV infection, cancer category, or broader pathological group.

From a clinical perspective, these findings emphasize that effective virological control and CD4+ T-cell recovery may not fully capture the cumulative effects of historical immunosuppression, oncogenic viral persistence, aging, and long-term immune dysregulation. Cancer prevention in PLWH should therefore remain integrated into long-term HIV care through established screening programs, vaccination against oncogenic viruses, management of modifiable risk factors, and surveillance for virus-associated malignancies.

At the same time, the composite inflammatory score offers a pragmatic framework for integrating routinely available markers of inflammatory, coagulation, and immune activity. Its association with current CD4+ T-cell count suggests that it may capture residual immune-inflammatory dysfunction. Its inability to distinguish ADCs from NADCs should not be interpreted as evidence that inflammation is biologically irrelevant; rather, it may indicate that conventional circulating markers are insufficiently sensitive to detect localized or cancer-specific immune-inflammatory processes. The score should therefore be considered an exploratory tool requiring validation. Longitudinal assessment may be particularly informative, as serial changes could better characterize residual immune dysfunction and its relationship with incident cancer, disease activity, treatment response, or prognosis.

Recent evidence further supports the relevance of persistent immune dysfunction and inflammaging in PLWH receiving effective antiretroviral therapy, as chronic inflammatory and immune alterations may persist despite virological control [24]. Large-scale evidence also confirms that cancer risk remains increased in PLWH and reflects the complex contribution of immunodeficiency and other cancer-related determinants [25]. Longitudinal measures of immune dysfunction, including CD4+ T-cell counts and the CD4/CD8 ratio, may provide additional information beyond single cross-sectional measurements when evaluating cancer susceptibility [26]. More recent data from virologically suppressed PLWH further indicate that the degree of immune recovery is associated with subsequent cancer risk [27,28].

This study has limitations inherent to its retrospective, single-center design, including limited subgroup sizes and incomplete availability of potential confounders such as acute infections, smoking, obesity, hepatitis coinfection, and active cancer treatment at the time of biomarker assessment. Inflammatory biomarkers were assessed cross-sectionally in 2024 rather than longitudinally before or at the time of cancer diagnosis. Consequently, the study cannot determine whether inflammation preceded cancer development, contributed to cancer susceptibility, or instead reflects current immune-inflammatory status, cancer-related factors, treatment effects, comorbidities, or intercurrent conditions. Moreover, the composite inflammatory score has not yet been externally validated, and alternative cutoffs or weighting schemes were not evaluated; the absence of an adjusted cancer-free comparison group precludes conclusions regarding its predictive value for cancer susceptibility. These findings should therefore be considered exploratory and warrant prospective validation.

Despite these limitations, this study extends the characterization of cancer in the Ferrara HIV cohort by integrating residual systemic inflammation through a composite biomarker approach. The findings support the feasibility of combining routinely available inflammatory, coagulation, and immune parameters to characterize systemic inflammatory burden in PLWH. Prospective studies incorporating longitudinal biomarker assessment and appropriate comparison groups are warranted to validate this approach and explore its potential contribution to cancer-risk characterization.

## 5. Conclusions

This study documents a substantial burden of cancer among PLWH receiving long-term antiretroviral therapy, with a cancer prevalence of 11.3% and a marked predominance of NADCs. Most cancer diagnoses occurred in older individuals and in those living with HIV for more than 10 years, highlighting the increasing impact of aging and cumulative exposure to conventional and HIV-related cancer risk factors.

Cancer occurred despite generally effective virological control and satisfactory current CD4+ T-cell counts, suggesting that conventional cross-sectional immunovirological parameters may not fully capture the cumulative oncological vulnerability of PLWH. The composite inflammatory score identified systemic inflammatory abnormalities in a subset of cancer diagnoses and was associated with immunological status, although it did not distinguish ADCs from NADCs. D-dimer elevation was descriptively more frequent among NADCs, supporting further investigation of the potential relevance of residual inflammatory and coagulation pathways in this population; however, this exploratory finding warrants confirmation in larger studies.

These findings reinforce the need to integrate cancer prevention and surveillance into long-term HIV care [29]. In clinical practice, the effectiveness of screening depends not only on the availability of recommended programs but also on their accessibility and suitability for PLWH. Stigma, fragmented care, and barriers to healthcare access may further affect screening uptake even when screening programs are available [30,31]. Cancer-prevention strategies should therefore consider individual risk profiles, HIV-related factors, and local healthcare characteristics alongside established screening recommendations. Integrating individualized cancer-prevention and screening pathways within HIV services may facilitate access, improve continuity and adherence to care, and enable closer surveillance of patients at greater oncological risk.

In this perspective, the local setting should not be viewed solely as a limitation of a single-center study, but also as an opportunity to develop prevention strategies tailored to the characteristics and needs of the population actually receiving care. Real-world cohorts can identify local patterns of cancer burden and support the development of feasible, patient-centered pathways that can subsequently be evaluated and adapted to other settings.

Because intensified oncological surveillance cannot be applied indiscriminately to all PLWH, simple and clinically accessible tools capable of identifying patients who may warrant greater attention could be particularly valuable. The composite inflammatory score explored in this study represents a pragmatic approach based on routinely available inflammatory, coagulation, and immune parameters. However, because the cancer-related analyses did not include a cancer-free PLWH comparison group, the present study cannot establish whether the observed inflammatory profiles are specifically associated with cancer susceptibility. Although it cannot currently be considered a predictive or screening tool, its simplicity and association with immunological status provide a rationale for investigating composite biomarker approaches as a potential component of multidimensional cancer-risk assessment. Combined with established risk factors and screening recommendations, such approaches could ultimately help identify PLWH who may benefit from targeted screening or closer oncological surveillance.

Prospective validation using longitudinal biomarker measurements and appropriate cancer-free comparison groups is therefore warranted. If validated, composite inflammatory scores could be readily incorporated into routine HIV care without requiring complex or costly investigations, supporting locally adapted and individualized cancer-prevention pathways and potentially facilitating earlier identification of patients requiring targeted screening or closer surveillance.

## Figures and Tables

**Table 1:** Demographic, clinical, immunovirological, and inflammatory characteristics of the study population (N = 566).

Characteristic	n/N (%)	Characteristic	n/N (%)
**Sex**		**CDC classification**	
Male	393/566 (69.4)	A1	40/566 (7.1)
Female	173/566 (30.6)	A2	183/566 (32.3)
**Geographical origin**		A3	33/566 (5.8)
Italian	470/566 (83.0)	B1	10/566 (1.8)
Male	347/566 (61.3)	B2	74/566 (13.1)
Female	123/566 (21.7)	B3	102/566 (18.0)
Non-Italian	96/566 (17.0)	C1	1/566 (0.2)
Male	46/566 (8.1)	C2	23/566 (4.0)
Female	50/566 (8.9)	C3	85/566 (15.0)
**Age, years**		Missing data	15/566 (2.7)
<30	10/566 (1.8)	**Plasma HIV RNA**	
30–45	104/566 (18.4)	Target not detected	288/566 (50.9)
46–65	356/566 (62.8)	<20 copies/mL	175/566 (30.9)
>65	96/566 (17.0)	20–49 copies/mL	32/566 (5.7)
**Duration of HIV infection**		≥50 copies/mL	41/566 (7.2)
<5 years	55/566 (9.7)	Missing data	30/566 (5.3)
5–10 years	77/566 (13.6)	**CD4+ T-cell count**	
>10 years	428/566 (75.6)	<200 cells/µL	27/566 (4.8)
Missing data	6/566 (1.1)	200–500 cells/µL	91/566 (16.1)
**IL-6**		>500 cells/µL	448/566 (79.1)
<6.4 pg/mL	477/566 (84.3)	**D-dimer**	
≥6.4 pg/mL	89/566 (15.7)	<0.5 µg/mL	454/566 (80.2)
**CRP**		≥0.5 µg/mL	107/566 (18.9)
<0.5 mg/dL	439/566 (77.6)	Missing data	5/566 (0.9)
≥0.5 mg/dL	126/566 (22.2)	**Monocyte count**	
Missing data	1/566 (0.2)	<200 cells/µL	4/566 (0.7)
		200–1000 cells/µL	549/566 (97.0)
		>1000 cells/µL	13/566 (2.3)

**Note:** Data are presented as n/N (%). **Abbreviations:** CDC, Centers for Disease Control and Prevention; CRP, C-reactive protein; HIV, human immunodeficiency virus; IL-6, interleukin-6.

**Table 2:** Distribution of cancer diagnoses classified as ADCs and NADCs.

Category	No. of Cases/Total (%)
Participants with a history of cancer	64/566 (11.3%)
Total cancer diagnoses	79/79 (100%)
ADC	23/79 (29.1%)
NADC	56/79 (70.9%)
**ADC distribution**	
Kaposi sarcoma	18/79 (22.8%)
Burkitt lymphoma	3/79 (3.8%)
Diffuse large B-cell lymphoma	2/79 (2.5%)
**NADC distribution**	
Skin cancers	25/79 (31.6%)
Head and neck cancers	9/79 (11.4%)
Thoracic cancers	8/79 (10.1%)
Genitourinary cancers	5/79 (6.3%)
Hematopoietic and lymphatic cancers	4/79 (5.1%)
Gastrointestinal cancers	3/79 (3.8%)
Neuroendocrine cancers	1/79 (1.3%)
Central nervous system cancers	1/79 (1.3%)

**Note:** Some participants had more than one malignancy; therefore, cancer prevalence was calculated at the patient level, whereas cancer distribution was calculated at the diagnosis level.

**Table 3:** Association between inflammatory score and demographic and clinical characteristics among cancer diagnoses (N = 79).

Characteristic	Absent/Mild Inflammation (Score 0–1)	Significant Inflammation (Score 2–4)	*p*-Value
**Overall**	65/79 (82.3%)	14/79 (17.7%)	
**Sex**			0.123
Male	47/79 (59.5%)	13/79 (16.5%)	
Female	18/79 (22.8%)	1/79 (1.3%)	
**Geographical Origin**			0.693
Italian	58/79 (73.4%)	13/79 (16.5%)	
Non-Italian	7/79 (8.9%)	1/79 (1.3%)	
**Age, years**			0.775
≤45	3/79 (3.8%)	1/79 (1.3%)	
46–65	40/79 (50.6%)	7/79 (8.9%)	
>65	22/79 (27.8%)	6/79 (7.6%)	
**Duration of HIV Infection**			0.103
≤10 years	8/79 (10.1%)	5/79 (6.3%)	
>10 years	57/79 (72.2%)	8/79 (10.1%)	
**CD4+ T-cell Count, cells/µL**			0.043
<200	3/79 (3.8%)	3/79 (3.8%)	
200–500	12/79 (15.2%)	5/79 (6.3%)	
>500	50/79 (63.3%)	6/79 (7.6%)	

**Note:** Data are presented as n/N (%). Associations were assessed using the Rao–Scott-adjusted chi-square test. Statistical significance was defined as a two-sided *p* < 0.05. Data on duration of HIV infection were missing for one cancer diagnosis. Analyses were conducted at the diagnosis level. Some participants contributed more than one cancer diagnosis, and within-patient correlation was accounted for using the patient identification code as the clustering variable.

**Table 4:** Association between inflammatory status and cancer category among cancer diagnoses (N = 79).

Neoplastic Category	Absent/Mild Inflammation (Score 0–1)	Significant Inflammation (Score 2–4)	*p*-Value
ADC	19/79 (24.1%)	4/79 (5.1%)	0.962
NADC	46/79 (58.2%)	10/79 (12.7%)	
Total	65/79 (82.3%)	14/79 (17.7%)	

**Table 5:** Analysis of pathological groups according to degree of inflammation among cancer diagnoses (N = 79).

Pathological Group	Total	Absent/Mild Inflammation (Score 0–1)	Significant Inflammation (Score 2–4)	*p*-Value
Lymphomas (Total)	8/79 (10.1%)	5/79 (6.3%)	3/79 (3.8%)	0.357
Kaposi Sarcoma	18/79 (22.8%)	16/79 (20.3%)	2/79 (2.5%)	
Skin Cancers (NADC)	25/79 (31.6%)	22/79 (27.8%)	3/79 (3.8%)	
Other NADC (visceral/CNS)	28/79 (35.4%)	22/79 (27.8%)	6/79 (7.6%)	

## Data Availability

The dataset generated and analyzed during the current study is not publicly available because it contains sensitive clinical information, but de-identified data are available from the corresponding author on reasonable request and subject to applicable ethical and institutional requirements.

## Abbreviations

The following abbreviations are used in this manuscript:

ADC	AIDS-defining cancer
AIDS	acquired immunodeficiency syndrome
cART	combination antiretroviral therapy
CDC	Centers for Disease Control and Prevention
CD4	cluster of differentiation 4
CRP	C-reactive protein
HIV	human immunodeficiency virus
IL-6	interleukin-6
NADC	non-AIDS-defining cancer
PLWH	people living with HIV
RNA	ribonucleic acid
sCD14	soluble CD14
sCD163	soluble CD163
